# (*E*)-*N*-Phenyl-*N*-(phenyl­carbamo­yl)-3-[prop­yl(tri­methyl­sil­yl)amino]­acryl­amide chloro­form hemisolvate

**DOI:** 10.1107/S2414314623001177

**Published:** 2023-02-17

**Authors:** Marcus Herbig, Uwe Böhme

**Affiliations:** a Institut für Anorganische Chemie, Technische Universität Bergakademie Freiberg, Leipziger Str. 29, 09599 Freiberg, Germany; University Koblenz-Landau, Germany

**Keywords:** crystal structure, organosilane, phenyl iso­cyanate, insertion

## Abstract

The title compound, C_22_H_29_N_3_O_2_Si·0.5CHCl_3_, crystallizes in the the triclinic space group *P*  with two host mol­ecules and one chloro­form mol­ecule in the asymmetric unit. The core of the mol­ecule consists of a urea unit bound to a 3-amino-acryloyl group. These units are almost planar in both mol­ecules [average deviation from plane of 0.05 (6) Å in mol­ecule *A* and 0.04 (5) Å in mol­ecule *B*]. The main difference between mol­ecules *A* and *B* involves the dihedral angles of the phenyl groups. One phenyl ring makes dihedral angles of 71.14 (6)° (mol­ecule *A*) and 82.81 (7)° (mol­ecule *B*) with respect to the core (C_4_N_3_O_2_) of the mol­ecule [14.56 (9)° (mol­ecule *A*) and 5.7 (1)° (mol­ecule *B*) for the other phenyl ring]. Another prominent feature is the intra­molecular N—H⋯O hydrogen bond present in both crystallographically independent mol­ecules.

## Abstract 2

The title compound, C_22_H_29_N_3_O_2_Si·0.5CHCl_3_, crystallizes in the the triclinic space group *P* 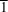 with two host mol­ecules and one chloro­form mol­ecule in the asymmetric unit. The core of the mol­ecule consists of a urea unit bound to a 3-amino-acryloyl group. An intra­molecular N—H⋯O hydrogen bond is present in both crystallographically independent mol­ecules.

## Graphical abstract

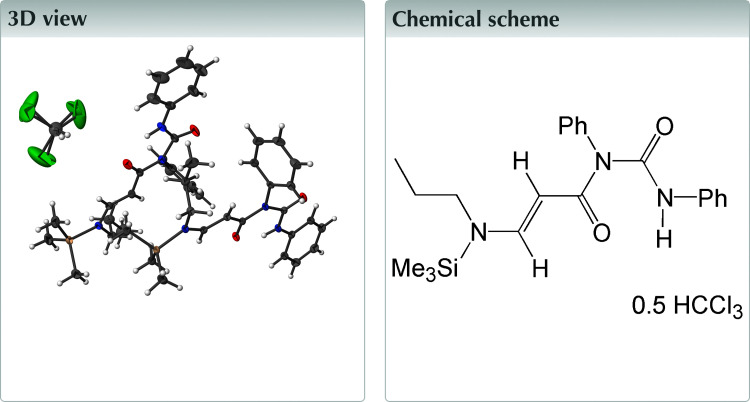

## Structure description

(*E*)-*N*-Phenyl-*N*-(phenyl­carbamo­yl)-3-[prop­yl(tri­methyl­sil­yl)amino]­acryl­amide is an insertion product from prop­yl(tri­methyl­sil­yl)[2-(tri­methyl­sil­yl)ethen­yl]amine and phenyl iso­cyanate. It was obtained in the course of our work on different types of silicon–nitro­gen compounds (Herbig *et al.*, 2019*a* ▸, 2021 ▸, 2022 ▸). Si—N bonds can be subjected to the insertion of different heteroallenes such as CO_2_ and iso­cyanates (Kraushaar *et al.*, 2012 ▸, 2014 ▸, 2017 ▸; Herbig *et al.*, 2018 ▸, 2019*b* ▸). In a continuation of our research in this area, the title compound was prepared and its crystal structure is reported here.

**Figure 1:**
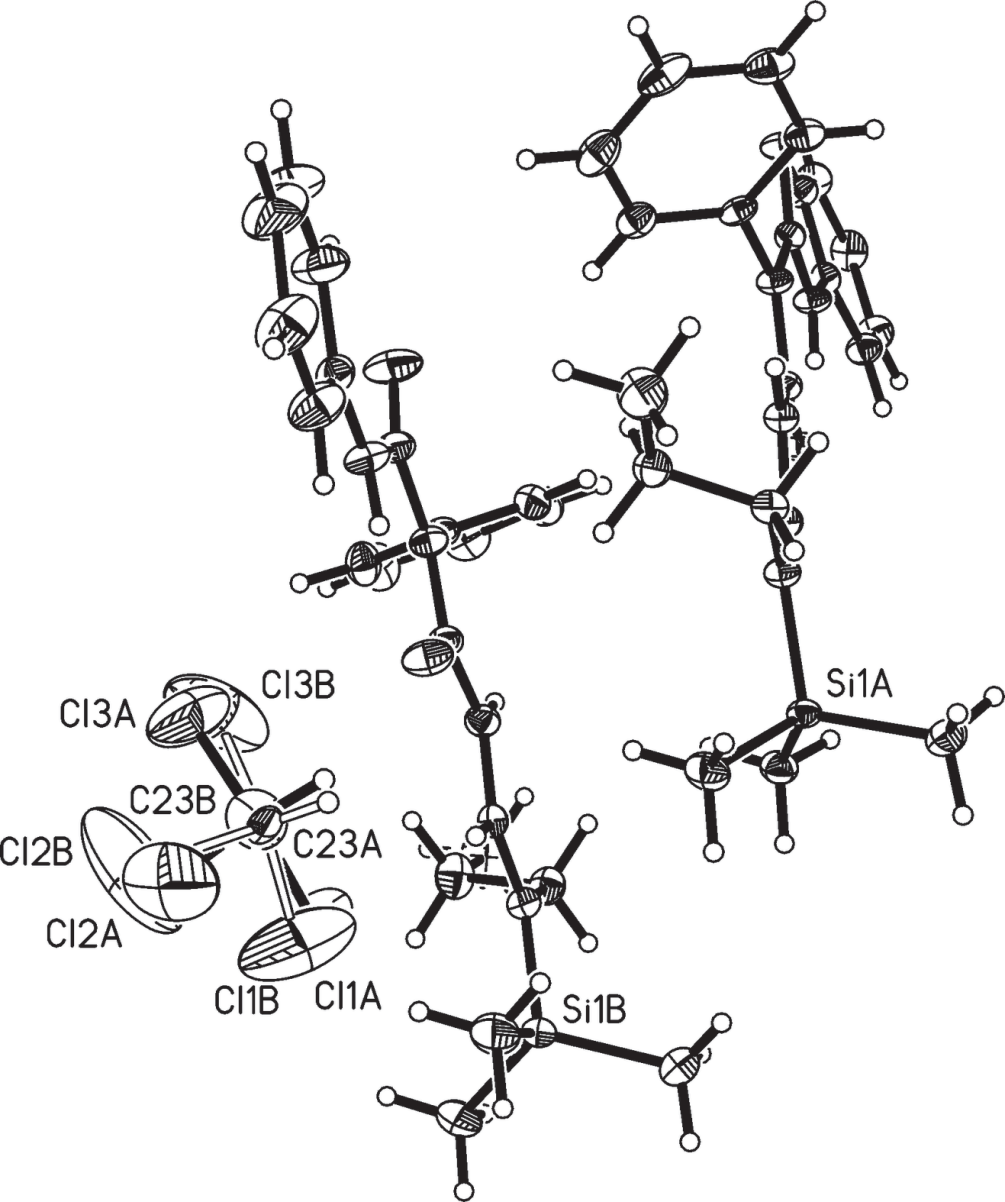
Asymmetric unit of the crystal structure including a disordered mol­ecule of chloro­form. Atomic displacement parameters are at the 50% probability level.

**Figure 2:**
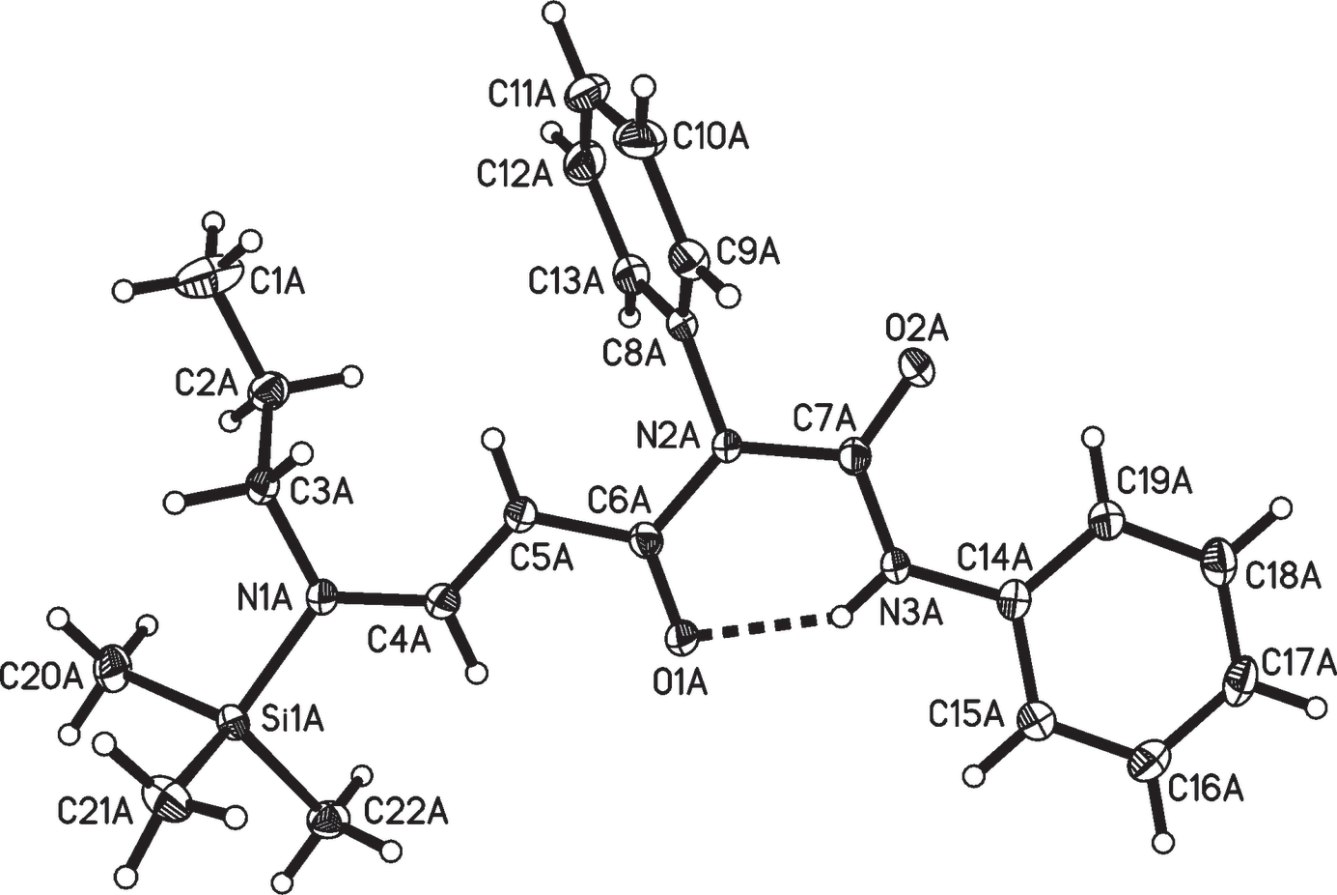
Diagram of mol­ecule *A* showing the atom-labelling scheme. Atomic displacement parameters are at the 50% probability level.

The title compound, C_22_H_29_N_3_O_2_Si·0.5 CHCl_3_, Fig. 1 ▸, crystallizes in the triclinic space group *P* 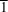 with two host mol­ecules (Figs. 2 ▸ and 3 ▸) and one chloro­form mol­ecule in the asymmetric unit. The core of the mol­ecule consists of a urea unit (N2, C7, O2, N3) linked to a 3-amino-acryloyl group (N1/C4–C6/O1). 3-Benz­yl­amino-2-cyano-*N*-[*N*-(2-fluoro­phen­yl) carbamo­yl]-3-(meth­yl­sulfan­yl)acryl­amide (Zhong *et al.*, 2011 ▸) is only one closely related acyclic structure in the CSD (Groom *et al.*, 2016 ▸). If the main structural elements of the title compound are allowed to occur in cyclic structures, purine derivatives are obtained, to which belongs for example, caffeine (Sutor, 1958 ▸). The formation of these cyclic structures requires a 180° rotation for the C5—C6 and C6—N2 bonds. Therefore, this structural relationship is not recognizable at first glance. There are about 1500 crystal structures of such purine derivatives.

The core of the mol­ecule formed by N1, C4–C6, O1, N2, C7, O2 and N3 is almost planar in both mol­ecules of the title compound [the average deviation from the plane is 0.05 (6) Å in mol­ecule *A* and 0.04 (5) Å in mol­ecule *B*] The planarity is presumably due to the conjugated system of double bonds. The C14–C19 phenyl rings in both mol­ecules are not coplanar to the core of the mol­ecules but adopt dihedral angles to the latter of 14.56 (9)° (mol­ecule *A*) and 5.7 (1)° (mol­ecule *B*). This small deviation from planarity still allows conjugation between the C14–C19 phenyl ring and the urea part of the mol­ecule.

The C8–C13 phenyl rings in both mol­ecules subtend dihedral angles of 71.14 (6)° (mol­ecule *A*) and 82.81 (7)° (mol­ecule *B*) with the core of the mol­ecule. This almost perpendicular conformation may be explained by the presence of the oxygen atom O2 in a vicinal position to the respective phenyl group.

An intra­molecular N3—H3⋯O1 hydrogen bond is present in both crystallographically independent mol­ecules (see Table 1 ▸). Another intra­molecular inter­action is present between the *ortho*-phenyl hydrogen atom H19 and O2 in both mol­ecules. The inter­action C9*A*—H9*A*⋯O1*A* represents an inter­molecular hydrogen bond.

The chloro­form solvent mol­ecule is disordered in the crystal structure with site occupation factors of 72.6:27.4%. C—H⋯π inter­actions are present between the chloro­form C—H bond and the centroid of the C14*A*–C19*A* phenyl ring (see Table 1 ▸).

## Synthesis and crystallization

**Figure 4:**
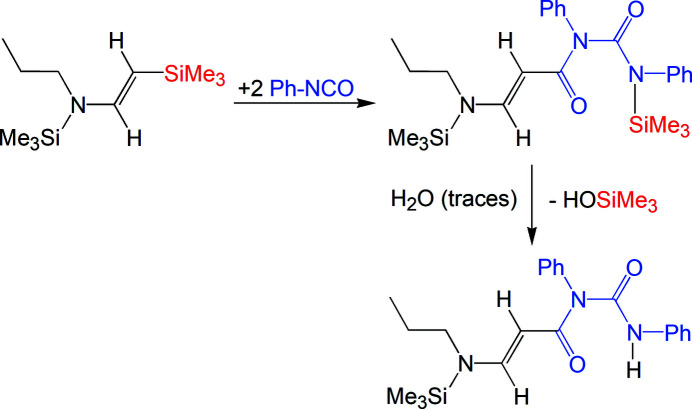
Proposed reaction scheme for the formation of the title compound.

(*E*)-*N*-phenyl-*N*-(phenyl­carbamo­yl)-3-[prop­yl(tri­methyl­sil­yl)amino])acryl­amide was obtained from the reaction of prop­yl(tri­methyl­sil­yl)[2–(tri­methyl­sil­yl)ethen­yl]amine and phenyl iso­cyanate. As shown in Fig. 4 ▸, a double insertion of Ph-NCO into the Si—C bond takes place (Herbig *et al.*, 2018 ▸). This reaction is possible due to the lability of bonds in the β-position of the enamine (Ozaki, 1972 ▸). Traces of water lead to the cleavage of one Si—C bond from the inter­mediate to yield the title compound.

To a solution of 0.46 g (2 mmol) prop­yl(tri­methyl­sil­yl)[2-(tri­methyl­sil­yl)ethen­yl]amine in 10 ml *n*-pentane were added dropwise 0.35 g (3 mmol) phenyl­iso­cyanate at 0°C. After standing for six days at room temperature, volatiles were removed under reduced pressure. Storing the product mixture for five years at −28°C yielded crystals suitable for single-crystal X-ray diffraction. No qu­anti­tative yield can be given here, since only a few crystals at the wall of the Schlenk tube were available. NMR spectroscopy showed that the batch product is a mixture of many components. Further purification of the product mixture was not successful.

## Refinement

Crystal data, data collection and structure refinement details are summarized in Table 2 ▸.

## Supplementary Material

Crystal structure: contains datablock(s) I. DOI: 10.1107/S2414314623001177/im4017sup1.cif

Structure factors: contains datablock(s) I. DOI: 10.1107/S2414314623001177/im4017Isup2.hkl

Supporting information file. DOI: 10.1107/S2414314623001177/im4017Isup3.cml

CCDC reference: 2240673

Additional supporting information:  crystallographic information; 3D view; checkCIF report

## Figures and Tables

**Figure 3:**
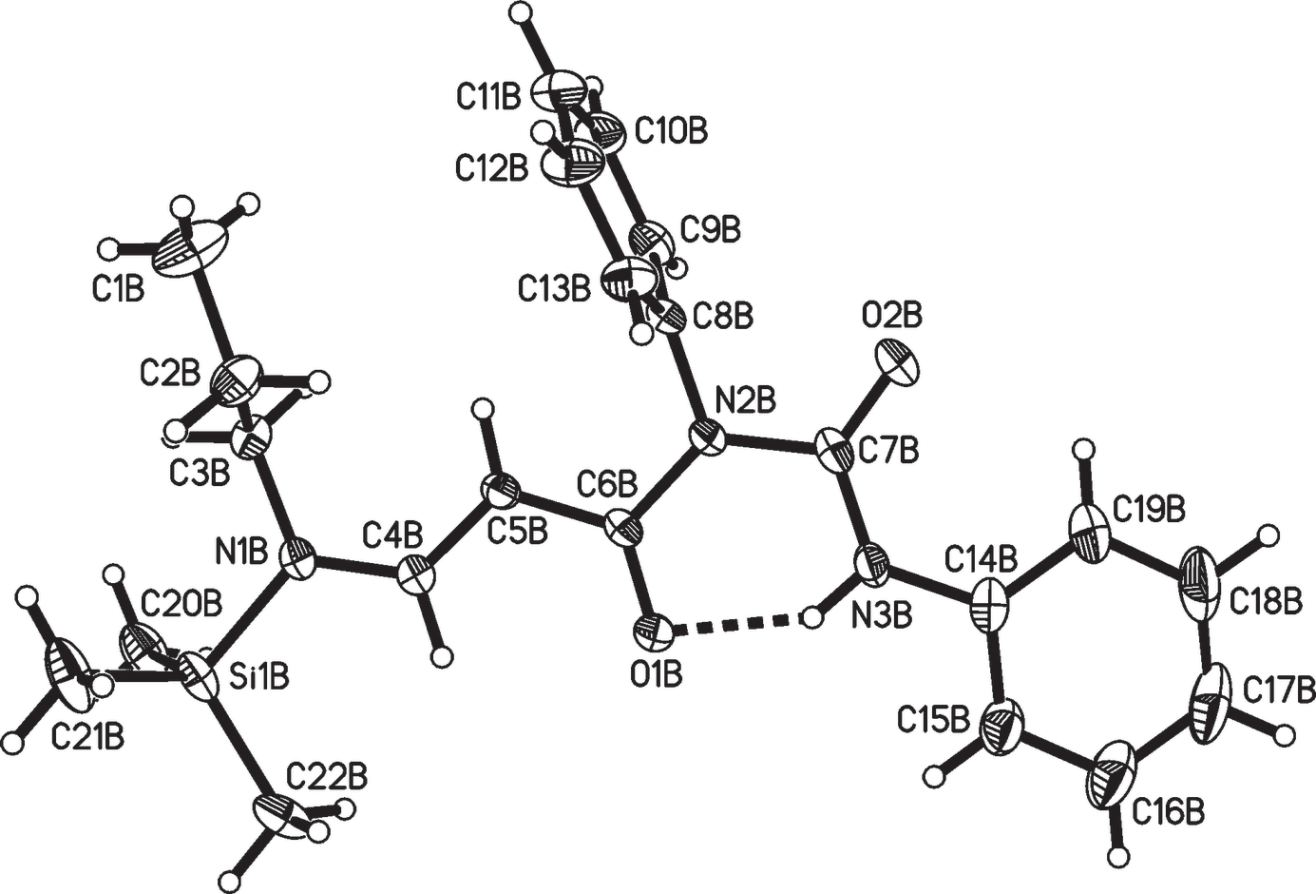
Diagram of mol­ecule *B* showing the atom-labelling scheme. Atomic displacement parameters are at the 50% probability level.

**Table 1:** Hydrogen-bond geometry (Å, °) *Cg1*is the centroid of the C14*A*–C19*A* phenyl ring.

*D*—H⋯*A*	*D*—H	H⋯*A*	*D*⋯*A*	*D*—H⋯*A*
N3*A*—H3*E*⋯O1*A*	0.85 (3)	1.83 (3)	2.564 (2)	144 (3)
N3*B*—H3*F*⋯O1*B*	0.90 (3)	1.80 (3)	2.584 (2)	144 (2)
C19*A*—H19*A*⋯O2*A*	0.95	2.29	2.880 (2)	120
C19*B*—H19*B*⋯O2*B*	0.95	2.30	2.900 (3)	120
C9*A*—H9*A*⋯O1*A* ^i^	0.95	2.46	3.378 (3)	161
C23*A*—H23*A*⋯*Cg*1^ii^	1.00	2.51	3.498 (8)	172
C23*B*—H23*B*⋯*Cg*1^ii^	1.00	2.47	3.43 (2)	161

Symmetry codes: (i) 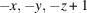 ; (ii) 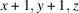 .

**Table 2:** Experimental details

Crystal data	
Chemical formula	2C_22_H_29_N_3_O_2_Si·CHCl_3_
*M*_r_	910.51
Crystal system, space group	Triclinic, *P* 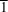
Temperature (K)	153
*a*, *b*, *c* (Å)	11.8904 (4), 12.5405 (4), 17.9387 (6)
α, β, γ (°)	107.849 (3), 95.849 (3), 101.372 (3)
*V* (Å^3^)	2458.07 (15)
*Z*	2
Radiation type	Mo *K*α
μ (mm^−1^)	0.28
Crystal size (mm)	0.45 × 0.40 × 0.15

Data collection	
Diffractometer	Stoe *IPDS* 2
Absorption correction	Integration (*X-RED*; Stoe, 2009 ▸)
*T*_min_, *T*_max_	0.817, 0.982
No. of measured, independent and observed [*I* > 2σ(*I*)] reflections	54315, 11291, 8839
*R*_int_	0.069
(sin θ/λ)_max_ (Å^−1^)	0.650

Refinement	
*R*[*F* ^2^ > 2σ(*F* ^2^)], *wR*(*F* ^2^), *S*	0.055, 0.123, 1.17
No. of reflections	11291
No. of parameters	611
No. of restraints	36
H-atom treatment	H atoms treated by a mixture of independent and constrained refinement
Δρ_max_, Δρ_min_ (e Å^−3^)	0.42, −0.42

Computer programs: *X-AREA* and *X-RED* (Stoe, 2009 ▸), *SHELXT* (Sheldrick, 2015*a* ▸), *SHELXL2018* (Sheldrick, 2015*b* ▸) and *ORTEP-3 for Windows* (Farrugia, 2012 ▸).

## full crystallographic data

### Crystal data

**Table:**

2C_22_H_29_N_3_O_2_Si·CHCl_3_	*Z* = 2
*M**_r_* = 910.51	*F*(000) = 964
Triclinic, *P*1	*D*_x_ = 1.230 Mg m^−^^3^
*a* = 11.8904 (4) Å	Mo *K*α radiation, λ = 0.71073 Å
*b* = 12.5405 (4) Å	Cell parameters from 54315 reflections
*c* = 17.9387 (6) Å	θ = 1.8–27.5°
α = 107.849 (3)°	µ = 0.28 mm^−^^1^
β = 95.849 (3)°	*T* = 153 K
γ = 101.372 (3)°	Prism, pale yellow
*V* = 2458.07 (15) Å^3^	0.45 × 0.40 × 0.15 mm

### Data collection

**Table:**

Stoe IPDS 2 diffractometer	11291 independent reflections
Radiation source: sealed X-ray tube, 12 x 0.4 mm long-fine focus	8839 reflections with *I* > 2σ(*I*)
Plane graphite monochromator	*R*_int_ = 0.069
Detector resolution: 6.67 pixels mm^-1^	θ_max_ = 27.5°, θ_min_ = 1.8°
rotation method scans	*h* = −14→15
Absorption correction: integration (X-RED; Stoe, 2009)	*k* = −16→16
*T*_min_ = 0.817, *T*_max_ = 0.982	*l* = −23→23
54315 measured reflections	

### Refinement

**Table:**

Refinement on *F*^2^	Hydrogen site location: mixed
Least-squares matrix: full	H atoms treated by a mixture of independent and constrained refinement
*R*[*F*^2^ > 2σ(*F*^2^)] = 0.055	*w* = 1/[σ^2^(*F*_o_^2^) + (0.0328*P*)^2^ + 2.1873*P*] where *P* = (*F*_o_^2^ + 2*F*_c_^2^)/3
*wR*(*F*^2^) = 0.123	(Δ/σ)_max_ = 0.001
*S* = 1.17	Δρ_max_ = 0.42 e Å^−^^3^
11291 reflections	Δρ_min_ = −0.42 e Å^−^^3^
611 parameters	Extinction correction: *SHELXL2017/1* (Sheldrick 2015b), Fc^*^=kFc[1+0.001xFc^2^λ^3^/sin(2θ)]^-1/4^
36 restraints	Extinction coefficient: 0.0038 (6)
Primary atom site location: structure-invariant direct methods	

### Special details

**Table:**

Geometry. All esds (except the esd in the dihedral angle between two l.s. planes) are estimated using the full covariance matrix. The cell esds are taken into account individually in the estimation of esds in distances, angles and torsion angles; correlations between esds in cell parameters are only used when they are defined by crystal symmetry. An approximate (isotropic) treatment of cell esds is used for estimating esds involving l.s. planes.
Refinement. Hydrogen atoms bonded to C were positioned geometrically and allowed to ride on their parent atoms, with C—H = 0.95 Å for *H*(phenyl), 0.99 Å for CH_2_, 0.98 Å for CH_3_ and 1.0 Å for *H*(chloroform). *U*_iso_(H) = *xU*_eq_(C), where *x* = 1.2 for *H*(phenyl), CH_2_, and *H*(chloroform) and 1.5 for CH_3_. Hydrogen atoms at nitrogen atoms N3A and N3B and at olefinic carbon atoms (C4A, C4B, C5A, and C5B) were localized from residual electron density maps and were freely refined.

### Fractional atomic coordinates and isotropic or equivalent isotropic displacement parameters (Å^2^)

**Table:**

	*x*	*y*	*z*	*U*_iso_*/*U*_eq_	Occ. (<1)
Si1A	0.53631 (4)	0.23095 (5)	0.59880 (3)	0.01628 (12)	
N1A	0.39528 (14)	0.25836 (14)	0.58740 (10)	0.0165 (3)	
C1A	0.3406 (3)	0.5463 (2)	0.58109 (17)	0.0417 (6)	
H1A	0.407277	0.567774	0.556240	0.063*	
H1B	0.331823	0.614774	0.622488	0.063*	
H1C	0.269735	0.514457	0.540719	0.063*	
C2A	0.36093 (19)	0.45635 (17)	0.61797 (13)	0.0224 (4)	
H2A	0.293221	0.433938	0.642521	0.027*	
H2B	0.430703	0.489557	0.660316	0.027*	
C3A	0.37822 (17)	0.35008 (17)	0.55549 (12)	0.0174 (4)	
H3A	0.309305	0.319135	0.512453	0.021*	
H3B	0.446877	0.373069	0.531950	0.021*	
C4A	0.30031 (17)	0.19110 (17)	0.59944 (11)	0.0164 (4)	
H4A	0.3146 (18)	0.1329 (18)	0.6216 (12)	0.011 (5)*	
C5A	0.18748 (16)	0.19702 (17)	0.58591 (12)	0.0164 (4)	
H5A	0.1675 (19)	0.2521 (19)	0.5644 (13)	0.017 (5)*	
C6A	0.10055 (16)	0.11577 (16)	0.60454 (12)	0.0164 (4)	
O1A	0.12410 (12)	0.03836 (12)	0.62846 (9)	0.0226 (3)	
N2A	−0.01519 (13)	0.12775 (14)	0.59446 (10)	0.0165 (3)	
C7A	−0.11611 (16)	0.04834 (16)	0.59948 (11)	0.0162 (4)	
O2A	−0.21194 (12)	0.06611 (13)	0.58615 (9)	0.0228 (3)	
N3A	−0.09445 (14)	−0.04211 (14)	0.61935 (11)	0.0185 (3)	
H3E	−0.023 (3)	−0.042 (2)	0.6274 (17)	0.042 (8)*	
C8A	−0.03738 (16)	0.23085 (17)	0.58167 (12)	0.0174 (4)	
C9A	−0.09394 (17)	0.22435 (19)	0.50850 (13)	0.0222 (4)	
H9A	−0.120708	0.152111	0.466889	0.027*	
C10A	−0.1111 (2)	0.3249 (2)	0.49667 (15)	0.0300 (5)	
H10A	−0.150059	0.321455	0.446758	0.036*	
C11A	−0.0715 (2)	0.4300 (2)	0.55748 (16)	0.0314 (5)	
H11A	−0.082837	0.498546	0.548986	0.038*	
C12A	−0.0155 (2)	0.43556 (19)	0.63054 (15)	0.0302 (5)	
H12A	0.011550	0.507836	0.672086	0.036*	
C13A	0.00126 (18)	0.33547 (18)	0.64313 (13)	0.0229 (4)	
H13A	0.038833	0.338711	0.693396	0.027*	
C14A	−0.17583 (17)	−0.13019 (17)	0.63154 (11)	0.0171 (4)	
C15A	−0.13700 (18)	−0.22580 (18)	0.63712 (13)	0.0222 (4)	
H15A	−0.059669	−0.230292	0.630321	0.027*	
C16A	−0.2103 (2)	−0.31462 (19)	0.65252 (14)	0.0266 (5)	
H16A	−0.182804	−0.379102	0.656646	0.032*	
C17A	−0.32377 (19)	−0.30915 (19)	0.66187 (14)	0.0265 (5)	
H17A	−0.374055	−0.369245	0.672895	0.032*	
C18A	−0.36262 (19)	−0.21519 (19)	0.65493 (13)	0.0252 (5)	
H18A	−0.440576	−0.211811	0.660471	0.030*	
C19A	−0.28983 (17)	−0.12550 (18)	0.64000 (12)	0.0204 (4)	
H19A	−0.317862	−0.061407	0.635603	0.024*	
C20A	0.64473 (18)	0.37213 (19)	0.64477 (14)	0.0264 (5)	
H20A	0.645650	0.416166	0.608067	0.040*	
H20B	0.722212	0.358824	0.655951	0.040*	
H20C	0.623401	0.415728	0.694479	0.040*	
C21A	0.5652 (2)	0.1556 (2)	0.49887 (13)	0.0276 (5)	
H21A	0.500390	0.088326	0.471175	0.041*	
H21B	0.637477	0.130151	0.504458	0.041*	
H21C	0.572966	0.208372	0.468161	0.041*	
C22A	0.53612 (18)	0.14171 (18)	0.66408 (13)	0.0223 (4)	
H22A	0.512616	0.180935	0.714115	0.033*	
H22B	0.614491	0.130352	0.675195	0.033*	
H22C	0.481136	0.066593	0.637482	0.033*	
Si1B	0.84710 (5)	0.32972 (6)	0.86670 (4)	0.02534 (15)	
N1B	0.69589 (15)	0.31525 (15)	0.86978 (10)	0.0202 (4)	
C1B	0.5352 (3)	0.1396 (3)	0.98522 (18)	0.0513 (8)	
H1D	0.462311	0.108730	0.946681	0.077*	
H1E	0.517577	0.160887	1.039283	0.077*	
H1F	0.580279	0.080708	0.977980	0.077*	
C2B	0.6055 (2)	0.2454 (2)	0.97240 (14)	0.0305 (5)	
H2C	0.679719	0.275875	1.010775	0.037*	
H2D	0.561513	0.306219	0.982118	0.037*	
C3B	0.63127 (19)	0.21576 (18)	0.88774 (13)	0.0239 (4)	
H3C	0.676916	0.156322	0.879165	0.029*	
H3D	0.556640	0.181901	0.849921	0.029*	
C4B	0.63751 (17)	0.39084 (18)	0.85520 (11)	0.0182 (4)	
H4B	0.684 (2)	0.456 (2)	0.8446 (14)	0.024 (6)*	
C5B	0.52209 (17)	0.38597 (18)	0.85359 (12)	0.0193 (4)	
H5B	0.471 (2)	0.327 (2)	0.8650 (14)	0.022 (6)*	
C6B	0.47745 (17)	0.47721 (18)	0.83768 (12)	0.0191 (4)	
O1B	0.53795 (13)	0.55653 (13)	0.82116 (10)	0.0271 (3)	
N2B	0.35896 (14)	0.47275 (15)	0.84306 (10)	0.0199 (4)	
C7B	0.29421 (18)	0.55001 (19)	0.82724 (13)	0.0232 (4)	
O2B	0.19006 (14)	0.53001 (16)	0.82805 (12)	0.0378 (4)	
N3B	0.35794 (16)	0.64190 (16)	0.81410 (11)	0.0243 (4)	
H3F	0.433 (2)	0.639 (2)	0.8122 (15)	0.029 (7)*	
C8B	0.29571 (17)	0.38468 (18)	0.86997 (13)	0.0204 (4)	
C9B	0.23157 (18)	0.28060 (19)	0.81549 (13)	0.0258 (5)	
H9B	0.221522	0.269170	0.760136	0.031*	
C10B	0.1821 (2)	0.1931 (2)	0.84293 (15)	0.0323 (5)	
H10B	0.137461	0.121378	0.806189	0.039*	
C11B	0.1977 (2)	0.2100 (2)	0.92381 (16)	0.0351 (6)	
H11B	0.164941	0.149412	0.942240	0.042*	
C12B	0.2608 (2)	0.3151 (2)	0.97764 (15)	0.0349 (6)	
H12B	0.270886	0.326955	1.033052	0.042*	
C13B	0.3092 (2)	0.4027 (2)	0.95046 (13)	0.0274 (5)	
H13B	0.351729	0.475352	0.987259	0.033*	
C14B	0.3177 (2)	0.7284 (2)	0.79240 (13)	0.0266 (5)	
C15B	0.4012 (2)	0.8081 (2)	0.77542 (16)	0.0367 (6)	
H15B	0.480185	0.803287	0.780883	0.044*	
C16B	0.3704 (3)	0.8943 (3)	0.75063 (18)	0.0486 (7)	
H16B	0.427997	0.947767	0.738593	0.058*	
C17B	0.2558 (3)	0.9025 (3)	0.74344 (19)	0.0558 (9)	
H17B	0.233786	0.960241	0.725253	0.067*	
C18B	0.1740 (3)	0.8260 (3)	0.7629 (2)	0.0550 (8)	
H18B	0.095737	0.833136	0.759378	0.066*	
C19B	0.2029 (2)	0.7385 (2)	0.78768 (17)	0.0409 (6)	
H19B	0.145420	0.686604	0.801103	0.049*	
C20B	0.8620 (2)	0.2053 (2)	0.78358 (15)	0.0356 (6)	
H20D	0.820354	0.133407	0.789350	0.053*	
H20E	0.944585	0.205739	0.784364	0.053*	
H20F	0.828826	0.210924	0.733008	0.053*	
C21B	0.9185 (2)	0.3294 (3)	0.96314 (16)	0.0446 (7)	
H21D	0.904945	0.393195	1.006273	0.067*	
H21E	1.002374	0.338944	0.963688	0.067*	
H21F	0.885862	0.256044	0.970580	0.067*	
C22B	0.9033 (2)	0.4663 (2)	0.84800 (16)	0.0373 (6)	
H22D	0.861469	0.463469	0.797199	0.056*	
H22E	0.986645	0.476221	0.845962	0.056*	
H22F	0.891493	0.531275	0.890969	0.056*	
C23A	0.8351 (6)	0.8733 (6)	0.8529 (5)	0.041 (3)	0.726 (13)
H23A	0.803377	0.848695	0.794825	0.049*	0.726 (13)
Cl1A	0.8931 (3)	0.7619 (2)	0.86680 (15)	0.0594 (7)	0.726 (13)
Cl2A	0.9415 (2)	0.9989 (2)	0.8746 (3)	0.0912 (11)	0.726 (13)
Cl3A	0.7188 (3)	0.8881 (3)	0.9013 (2)	0.0756 (9)	0.726 (13)
C23B	0.8402 (11)	0.8686 (11)	0.8482 (12)	0.030 (7)	0.274 (13)
H23B	0.831173	0.856202	0.789897	0.036*	0.274 (13)
Cl1B	0.9176 (14)	0.7799 (13)	0.8780 (9)	0.138 (5)	0.274 (13)
Cl2B	0.9224 (13)	1.0053 (8)	0.9078 (10)	0.154 (5)	0.274 (13)
Cl3B	0.7109 (8)	0.8654 (13)	0.8849 (8)	0.123 (4)	0.274 (13)

### Atomic displacement parameters (Å^2^)

**Table:**

	*U*^11^	*U*^22^	*U*^33^	*U*^12^	*U*^13^	*U*^23^
Si1A	0.0115 (2)	0.0170 (3)	0.0201 (3)	0.0027 (2)	0.0024 (2)	0.0063 (2)
N1A	0.0131 (7)	0.0162 (8)	0.0216 (8)	0.0025 (6)	0.0036 (6)	0.0087 (7)
C1A	0.0563 (17)	0.0250 (12)	0.0428 (15)	0.0159 (12)	−0.0049 (13)	0.0104 (11)
C2A	0.0225 (10)	0.0176 (10)	0.0257 (11)	0.0038 (8)	0.0019 (8)	0.0068 (8)
C3A	0.0163 (9)	0.0190 (10)	0.0195 (10)	0.0036 (7)	0.0046 (8)	0.0100 (8)
C4A	0.0161 (9)	0.0153 (9)	0.0176 (9)	0.0033 (7)	0.0026 (7)	0.0056 (8)
C5A	0.0148 (9)	0.0160 (9)	0.0195 (10)	0.0027 (7)	0.0032 (7)	0.0082 (8)
C6A	0.0132 (9)	0.0157 (9)	0.0186 (9)	0.0023 (7)	0.0016 (7)	0.0044 (7)
O1A	0.0139 (7)	0.0202 (7)	0.0379 (9)	0.0036 (6)	0.0030 (6)	0.0168 (7)
N2A	0.0113 (7)	0.0148 (8)	0.0251 (9)	0.0023 (6)	0.0032 (6)	0.0098 (7)
C7A	0.0142 (9)	0.0155 (9)	0.0179 (9)	0.0017 (7)	0.0029 (7)	0.0054 (7)
O2A	0.0130 (7)	0.0254 (8)	0.0340 (8)	0.0053 (6)	0.0045 (6)	0.0152 (7)
N3A	0.0111 (8)	0.0170 (8)	0.0291 (9)	0.0019 (6)	0.0037 (7)	0.0110 (7)
C8A	0.0130 (9)	0.0160 (9)	0.0274 (10)	0.0053 (7)	0.0081 (8)	0.0106 (8)
C9A	0.0178 (10)	0.0253 (11)	0.0274 (11)	0.0071 (8)	0.0064 (8)	0.0122 (9)
C10A	0.0296 (12)	0.0371 (13)	0.0365 (13)	0.0172 (10)	0.0105 (10)	0.0235 (11)
C11A	0.0334 (12)	0.0248 (11)	0.0511 (15)	0.0158 (10)	0.0216 (11)	0.0241 (11)
C12A	0.0302 (12)	0.0193 (11)	0.0428 (14)	0.0068 (9)	0.0150 (10)	0.0097 (10)
C13A	0.0183 (10)	0.0208 (10)	0.0305 (11)	0.0046 (8)	0.0075 (8)	0.0090 (9)
C14A	0.0164 (9)	0.0174 (9)	0.0161 (9)	0.0008 (7)	0.0028 (7)	0.0056 (8)
C15A	0.0188 (10)	0.0212 (10)	0.0278 (11)	0.0051 (8)	0.0051 (8)	0.0093 (9)
C16A	0.0290 (11)	0.0195 (10)	0.0336 (12)	0.0051 (9)	0.0072 (9)	0.0121 (9)
C17A	0.0263 (11)	0.0205 (10)	0.0309 (12)	−0.0030 (8)	0.0083 (9)	0.0102 (9)
C18A	0.0197 (10)	0.0265 (11)	0.0281 (11)	0.0008 (8)	0.0074 (9)	0.0092 (9)
C19A	0.0176 (9)	0.0193 (10)	0.0241 (10)	0.0035 (8)	0.0048 (8)	0.0073 (8)
C20A	0.0175 (10)	0.0249 (11)	0.0327 (12)	−0.0018 (8)	0.0010 (9)	0.0092 (9)
C21A	0.0248 (11)	0.0355 (13)	0.0260 (11)	0.0139 (10)	0.0080 (9)	0.0098 (10)
C22A	0.0214 (10)	0.0213 (10)	0.0243 (11)	0.0063 (8)	−0.0001 (8)	0.0083 (9)
Si1B	0.0169 (3)	0.0309 (3)	0.0231 (3)	0.0092 (2)	−0.0008 (2)	0.0010 (3)
N1B	0.0185 (8)	0.0220 (9)	0.0203 (9)	0.0074 (7)	0.0031 (7)	0.0058 (7)
C1B	0.074 (2)	0.0396 (16)	0.0452 (17)	0.0053 (15)	0.0231 (16)	0.0218 (13)
C2B	0.0401 (13)	0.0290 (12)	0.0249 (11)	0.0083 (10)	0.0080 (10)	0.0118 (10)
C3B	0.0278 (11)	0.0218 (10)	0.0241 (11)	0.0078 (9)	0.0049 (9)	0.0090 (9)
C4B	0.0189 (9)	0.0197 (10)	0.0146 (9)	0.0052 (8)	0.0024 (7)	0.0036 (8)
C5B	0.0160 (9)	0.0213 (10)	0.0206 (10)	0.0033 (8)	0.0035 (8)	0.0075 (8)
C6B	0.0148 (9)	0.0238 (10)	0.0188 (9)	0.0055 (8)	0.0042 (7)	0.0066 (8)
O1B	0.0196 (7)	0.0297 (8)	0.0401 (9)	0.0077 (6)	0.0108 (7)	0.0203 (7)
N2B	0.0152 (8)	0.0230 (9)	0.0241 (9)	0.0059 (7)	0.0060 (7)	0.0098 (7)
C7B	0.0183 (10)	0.0303 (11)	0.0230 (10)	0.0105 (8)	0.0045 (8)	0.0085 (9)
O2B	0.0182 (8)	0.0448 (10)	0.0601 (12)	0.0131 (7)	0.0119 (8)	0.0260 (9)
N3B	0.0199 (9)	0.0298 (10)	0.0305 (10)	0.0128 (8)	0.0085 (8)	0.0150 (8)
C8B	0.0142 (9)	0.0236 (10)	0.0251 (10)	0.0054 (8)	0.0067 (8)	0.0092 (8)
C9B	0.0201 (10)	0.0291 (12)	0.0243 (11)	0.0051 (9)	0.0017 (8)	0.0047 (9)
C10B	0.0238 (11)	0.0232 (11)	0.0419 (14)	−0.0014 (9)	0.0011 (10)	0.0055 (10)
C11B	0.0311 (13)	0.0322 (13)	0.0451 (15)	0.0027 (10)	0.0123 (11)	0.0186 (11)
C12B	0.0376 (13)	0.0410 (14)	0.0276 (12)	0.0034 (11)	0.0111 (10)	0.0157 (11)
C13B	0.0299 (12)	0.0269 (11)	0.0223 (11)	0.0020 (9)	0.0073 (9)	0.0060 (9)
C14B	0.0331 (12)	0.0293 (12)	0.0206 (10)	0.0166 (10)	0.0038 (9)	0.0072 (9)
C15B	0.0431 (14)	0.0407 (14)	0.0422 (14)	0.0221 (12)	0.0183 (12)	0.0252 (12)
C16B	0.071 (2)	0.0455 (16)	0.0507 (17)	0.0310 (15)	0.0246 (15)	0.0307 (14)
C17B	0.080 (2)	0.0543 (19)	0.0565 (19)	0.0459 (18)	0.0158 (17)	0.0323 (16)
C18B	0.0489 (18)	0.0583 (19)	0.069 (2)	0.0346 (16)	0.0026 (16)	0.0257 (17)
C19B	0.0315 (13)	0.0446 (15)	0.0537 (17)	0.0200 (12)	0.0046 (12)	0.0204 (13)
C20B	0.0276 (12)	0.0426 (14)	0.0321 (13)	0.0156 (11)	0.0061 (10)	0.0014 (11)
C21B	0.0331 (14)	0.0607 (18)	0.0323 (14)	0.0209 (13)	−0.0098 (11)	0.0036 (13)
C22B	0.0191 (11)	0.0428 (15)	0.0422 (15)	0.0002 (10)	0.0059 (10)	0.0075 (12)
C23A	0.050 (5)	0.043 (5)	0.038 (4)	0.007 (4)	0.008 (4)	0.028 (4)
Cl1A	0.0840 (13)	0.0599 (11)	0.0711 (12)	0.0407 (9)	0.0421 (9)	0.0503 (8)
Cl2A	0.0638 (11)	0.0482 (10)	0.156 (3)	−0.0127 (8)	0.0034 (13)	0.0478 (13)
Cl3A	0.0710 (16)	0.1108 (16)	0.0802 (18)	0.0586 (14)	0.0431 (13)	0.0494 (13)
C23B	0.010 (7)	0.019 (9)	0.046 (12)	−0.001 (6)	0.006 (7)	−0.010 (7)
Cl1B	0.115 (7)	0.169 (9)	0.192 (10)	0.101 (7)	0.059 (6)	0.097 (7)
Cl2B	0.160 (8)	0.101 (5)	0.133 (8)	−0.081 (5)	−0.054 (6)	0.039 (5)
Cl3B	0.046 (3)	0.248 (10)	0.062 (4)	0.002 (4)	0.015 (2)	0.056 (5)

### Geometric parameters (Å, º)

**Table:**

Si1A—N1A	1.7811 (17)	N1B—C4B	1.350 (3)
Si1A—C22A	1.851 (2)	N1B—C3B	1.473 (3)
Si1A—C21A	1.855 (2)	C1B—C2B	1.518 (3)
Si1A—C20A	1.861 (2)	C1B—H1D	0.9800
N1A—C4A	1.349 (2)	C1B—H1E	0.9800
N1A—C3A	1.471 (2)	C1B—H1F	0.9800
C1A—C2A	1.517 (3)	C2B—C3B	1.527 (3)
C1A—H1A	0.9800	C2B—H2C	0.9900
C1A—H1B	0.9800	C2B—H2D	0.9900
C1A—H1C	0.9800	C3B—H3C	0.9900
C2A—C3A	1.521 (3)	C3B—H3D	0.9900
C2A—H2A	0.9900	C4B—C5B	1.359 (3)
C2A—H2B	0.9900	C4B—H4B	0.97 (2)
C3A—H3A	0.9900	C5B—C6B	1.443 (3)
C3A—H3B	0.9900	C5B—H5B	0.95 (2)
C4A—C5A	1.360 (3)	C6B—O1B	1.240 (2)
C4A—H4A	0.97 (2)	C6B—N2B	1.413 (2)
C5A—C6A	1.443 (3)	N2B—C7B	1.425 (3)
C5A—H5A	0.94 (2)	N2B—C8B	1.446 (3)
C6A—O1A	1.242 (2)	C7B—O2B	1.217 (3)
C6A—N2A	1.413 (2)	C7B—N3B	1.348 (3)
N2A—C7A	1.430 (2)	N3B—C14B	1.409 (3)
N2A—C8A	1.448 (2)	N3B—H3F	0.90 (3)
C7A—O2A	1.218 (2)	C8B—C13B	1.378 (3)
C7A—N3A	1.351 (2)	C8B—C9B	1.383 (3)
N3A—C14A	1.405 (2)	C9B—C10B	1.388 (3)
N3A—H3E	0.85 (3)	C9B—H9B	0.9500
C8A—C9A	1.384 (3)	C10B—C11B	1.388 (4)
C8A—C13A	1.386 (3)	C10B—H10B	0.9500
C9A—C10A	1.390 (3)	C11B—C12B	1.383 (4)
C9A—H9A	0.9500	C11B—H11B	0.9500
C10A—C11A	1.385 (4)	C12B—C13B	1.383 (3)
C10A—H10A	0.9500	C12B—H12B	0.9500
C11A—C12A	1.383 (4)	C13B—H13B	0.9500
C11A—H11A	0.9500	C14B—C15B	1.390 (3)
C12A—C13A	1.389 (3)	C14B—C19B	1.391 (3)
C12A—H12A	0.9500	C15B—C16B	1.385 (3)
C13A—H13A	0.9500	C15B—H15B	0.9500
C14A—C19A	1.390 (3)	C16B—C17B	1.382 (4)
C14A—C15A	1.394 (3)	C16B—H16B	0.9500
C15A—C16A	1.390 (3)	C17B—C18B	1.377 (5)
C15A—H15A	0.9500	C17B—H17B	0.9500
C16A—C17A	1.389 (3)	C18B—C19B	1.391 (4)
C16A—H16A	0.9500	C18B—H18B	0.9500
C17A—C18A	1.382 (3)	C19B—H19B	0.9500
C17A—H17A	0.9500	C20B—H20D	0.9800
C18A—C19A	1.391 (3)	C20B—H20E	0.9800
C18A—H18A	0.9500	C20B—H20F	0.9800
C19A—H19A	0.9500	C21B—H21D	0.9800
C20A—H20A	0.9800	C21B—H21E	0.9800
C20A—H20B	0.9800	C21B—H21F	0.9800
C20A—H20C	0.9800	C22B—H22D	0.9800
C21A—H21A	0.9800	C22B—H22E	0.9800
C21A—H21B	0.9800	C22B—H22F	0.9800
C21A—H21C	0.9800	C23A—Cl3A	1.717 (7)
C22A—H22A	0.9800	C23A—Cl2A	1.724 (6)
C22A—H22B	0.9800	C23A—Cl1A	1.745 (6)
C22A—H22C	0.9800	C23A—H23A	1.0000
Si1B—N1B	1.7794 (18)	C23B—Cl3B	1.731 (13)
Si1B—C21B	1.849 (3)	C23B—Cl2B	1.743 (13)
Si1B—C20B	1.851 (2)	C23B—Cl1B	1.744 (13)
Si1B—C22B	1.854 (3)	C23B—H23B	1.0000
			
N1A—Si1A—C22A	107.35 (9)	C4B—N1B—C3B	118.60 (17)
N1A—Si1A—C21A	108.35 (9)	C4B—N1B—Si1B	122.71 (15)
C22A—Si1A—C21A	111.95 (10)	C3B—N1B—Si1B	118.69 (14)
N1A—Si1A—C20A	108.14 (9)	C2B—C1B—H1D	109.5
C22A—Si1A—C20A	111.09 (10)	C2B—C1B—H1E	109.5
C21A—Si1A—C20A	109.82 (11)	H1D—C1B—H1E	109.5
C4A—N1A—C3A	117.89 (16)	C2B—C1B—H1F	109.5
C4A—N1A—Si1A	122.62 (13)	H1D—C1B—H1F	109.5
C3A—N1A—Si1A	119.17 (12)	H1E—C1B—H1F	109.5
C2A—C1A—H1A	109.5	C1B—C2B—C3B	110.7 (2)
C2A—C1A—H1B	109.5	C1B—C2B—H2C	109.5
H1A—C1A—H1B	109.5	C3B—C2B—H2C	109.5
C2A—C1A—H1C	109.5	C1B—C2B—H2D	109.5
H1A—C1A—H1C	109.5	C3B—C2B—H2D	109.5
H1B—C1A—H1C	109.5	H2C—C2B—H2D	108.1
C1A—C2A—C3A	110.74 (19)	N1B—C3B—C2B	113.99 (18)
C1A—C2A—H2A	109.5	N1B—C3B—H3C	108.8
C3A—C2A—H2A	109.5	C2B—C3B—H3C	108.8
C1A—C2A—H2B	109.5	N1B—C3B—H3D	108.8
C3A—C2A—H2B	109.5	C2B—C3B—H3D	108.8
H2A—C2A—H2B	108.1	H3C—C3B—H3D	107.6
N1A—C3A—C2A	113.24 (16)	N1B—C4B—C5B	127.4 (2)
N1A—C3A—H3A	108.9	N1B—C4B—H4B	115.3 (14)
C2A—C3A—H3A	108.9	C5B—C4B—H4B	117.3 (14)
N1A—C3A—H3B	108.9	C4B—C5B—C6B	118.43 (19)
C2A—C3A—H3B	108.9	C4B—C5B—H5B	122.5 (14)
H3A—C3A—H3B	107.7	C6B—C5B—H5B	119.0 (14)
N1A—C4A—C5A	127.93 (18)	O1B—C6B—N2B	120.82 (18)
N1A—C4A—H4A	115.9 (13)	O1B—C6B—C5B	123.17 (18)
C5A—C4A—H4A	116.2 (13)	N2B—C6B—C5B	116.00 (17)
C4A—C5A—C6A	117.71 (18)	C6B—N2B—C7B	126.47 (17)
C4A—C5A—H5A	120.5 (14)	C6B—N2B—C8B	118.35 (16)
C6A—C5A—H5A	121.8 (14)	C7B—N2B—C8B	115.13 (16)
O1A—C6A—N2A	120.42 (17)	O2B—C7B—N3B	126.1 (2)
O1A—C6A—C5A	122.79 (17)	O2B—C7B—N2B	119.1 (2)
N2A—C6A—C5A	116.79 (17)	N3B—C7B—N2B	114.74 (18)
C6A—N2A—C7A	125.79 (16)	C7B—N3B—C14B	127.68 (19)
C6A—N2A—C8A	119.34 (15)	C7B—N3B—H3F	113.3 (16)
C7A—N2A—C8A	114.80 (15)	C14B—N3B—H3F	118.3 (16)
O2A—C7A—N3A	125.61 (18)	C13B—C8B—C9B	120.9 (2)
O2A—C7A—N2A	119.39 (17)	C13B—C8B—N2B	118.55 (19)
N3A—C7A—N2A	114.99 (16)	C9B—C8B—N2B	120.27 (19)
C7A—N3A—C14A	127.20 (17)	C8B—C9B—C10B	119.0 (2)
C7A—N3A—H3E	114.3 (19)	C8B—C9B—H9B	120.5
C14A—N3A—H3E	118.4 (19)	C10B—C9B—H9B	120.5
C9A—C8A—C13A	121.06 (19)	C11B—C10B—C9B	120.3 (2)
C9A—C8A—N2A	120.05 (18)	C11B—C10B—H10B	119.9
C13A—C8A—N2A	118.87 (18)	C9B—C10B—H10B	119.9
C8A—C9A—C10A	119.1 (2)	C12B—C11B—C10B	120.1 (2)
C8A—C9A—H9A	120.4	C12B—C11B—H11B	120.0
C10A—C9A—H9A	120.4	C10B—C11B—H11B	120.0
C11A—C10A—C9A	120.2 (2)	C11B—C12B—C13B	119.7 (2)
C11A—C10A—H10A	119.9	C11B—C12B—H12B	120.1
C9A—C10A—H10A	119.9	C13B—C12B—H12B	120.1
C12A—C11A—C10A	120.3 (2)	C8B—C13B—C12B	120.0 (2)
C12A—C11A—H11A	119.9	C8B—C13B—H13B	120.0
C10A—C11A—H11A	119.9	C12B—C13B—H13B	120.0
C11A—C12A—C13A	120.0 (2)	C15B—C14B—C19B	119.6 (2)
C11A—C12A—H12A	120.0	C15B—C14B—N3B	115.8 (2)
C13A—C12A—H12A	120.0	C19B—C14B—N3B	124.6 (2)
C8A—C13A—C12A	119.4 (2)	C16B—C15B—C14B	120.7 (3)
C8A—C13A—H13A	120.3	C16B—C15B—H15B	119.7
C12A—C13A—H13A	120.3	C14B—C15B—H15B	119.7
C19A—C14A—C15A	119.24 (18)	C17B—C16B—C15B	119.9 (3)
C19A—C14A—N3A	123.88 (18)	C17B—C16B—H16B	120.1
C15A—C14A—N3A	116.86 (17)	C15B—C16B—H16B	120.1
C16A—C15A—C14A	120.64 (19)	C18B—C17B—C16B	119.3 (3)
C16A—C15A—H15A	119.7	C18B—C17B—H17B	120.4
C14A—C15A—H15A	119.7	C16B—C17B—H17B	120.4
C17A—C16A—C15A	120.0 (2)	C17B—C18B—C19B	121.7 (3)
C17A—C16A—H16A	120.0	C17B—C18B—H18B	119.1
C15A—C16A—H16A	120.0	C19B—C18B—H18B	119.1
C18A—C17A—C16A	119.18 (19)	C18B—C19B—C14B	118.7 (3)
C18A—C17A—H17A	120.4	C18B—C19B—H19B	120.7
C16A—C17A—H17A	120.4	C14B—C19B—H19B	120.7
C17A—C18A—C19A	121.3 (2)	Si1B—C20B—H20D	109.5
C17A—C18A—H18A	119.4	Si1B—C20B—H20E	109.5
C19A—C18A—H18A	119.4	H20D—C20B—H20E	109.5
C14A—C19A—C18A	119.61 (19)	Si1B—C20B—H20F	109.5
C14A—C19A—H19A	120.2	H20D—C20B—H20F	109.5
C18A—C19A—H19A	120.2	H20E—C20B—H20F	109.5
Si1A—C20A—H20A	109.5	Si1B—C21B—H21D	109.5
Si1A—C20A—H20B	109.5	Si1B—C21B—H21E	109.5
H20A—C20A—H20B	109.5	H21D—C21B—H21E	109.5
Si1A—C20A—H20C	109.5	Si1B—C21B—H21F	109.5
H20A—C20A—H20C	109.5	H21D—C21B—H21F	109.5
H20B—C20A—H20C	109.5	H21E—C21B—H21F	109.5
Si1A—C21A—H21A	109.5	Si1B—C22B—H22D	109.5
Si1A—C21A—H21B	109.5	Si1B—C22B—H22E	109.5
H21A—C21A—H21B	109.5	H22D—C22B—H22E	109.5
Si1A—C21A—H21C	109.5	Si1B—C22B—H22F	109.5
H21A—C21A—H21C	109.5	H22D—C22B—H22F	109.5
H21B—C21A—H21C	109.5	H22E—C22B—H22F	109.5
Si1A—C22A—H22A	109.5	Cl3A—C23A—Cl2A	114.7 (4)
Si1A—C22A—H22B	109.5	Cl3A—C23A—Cl1A	111.0 (3)
H22A—C22A—H22B	109.5	Cl2A—C23A—Cl1A	111.6 (4)
Si1A—C22A—H22C	109.5	Cl3A—C23A—H23A	106.3
H22A—C22A—H22C	109.5	Cl2A—C23A—H23A	106.3
H22B—C22A—H22C	109.5	Cl1A—C23A—H23A	106.3
N1B—Si1B—C21B	107.54 (11)	Cl3B—C23B—Cl2B	100.9 (10)
N1B—Si1B—C20B	107.58 (10)	Cl3B—C23B—Cl1B	111.2 (11)
C21B—Si1B—C20B	111.25 (13)	Cl2B—C23B—Cl1B	101.4 (10)
N1B—Si1B—C22B	107.87 (10)	Cl3B—C23B—H23B	113.9
C21B—Si1B—C22B	112.33 (13)	Cl2B—C23B—H23B	113.9
C20B—Si1B—C22B	110.07 (13)	Cl1B—C23B—H23B	113.9
			
C22A—Si1A—N1A—C4A	−21.84 (18)	C21B—Si1B—N1B—C4B	125.21 (18)
C21A—Si1A—N1A—C4A	99.24 (17)	C20B—Si1B—N1B—C4B	−114.87 (18)
C20A—Si1A—N1A—C4A	−141.78 (16)	C22B—Si1B—N1B—C4B	3.8 (2)
C22A—Si1A—N1A—C3A	164.85 (14)	C21B—Si1B—N1B—C3B	−55.77 (19)
C21A—Si1A—N1A—C3A	−74.06 (17)	C20B—Si1B—N1B—C3B	64.16 (18)
C20A—Si1A—N1A—C3A	44.92 (17)	C22B—Si1B—N1B—C3B	−177.14 (16)
C4A—N1A—C3A—C2A	78.7 (2)	C4B—N1B—C3B—C2B	−77.2 (2)
Si1A—N1A—C3A—C2A	−107.67 (17)	Si1B—N1B—C3B—C2B	103.69 (19)
C1A—C2A—C3A—N1A	−178.50 (18)	C1B—C2B—C3B—N1B	178.0 (2)
C3A—N1A—C4A—C5A	−0.2 (3)	C3B—N1B—C4B—C5B	−1.2 (3)
Si1A—N1A—C4A—C5A	−173.63 (17)	Si1B—N1B—C4B—C5B	177.81 (17)
N1A—C4A—C5A—C6A	−179.24 (19)	N1B—C4B—C5B—C6B	179.22 (19)
C4A—C5A—C6A—O1A	−3.5 (3)	C4B—C5B—C6B—O1B	4.0 (3)
C4A—C5A—C6A—N2A	176.38 (18)	C4B—C5B—C6B—N2B	−175.07 (18)
O1A—C6A—N2A—C7A	−8.7 (3)	O1B—C6B—N2B—C7B	3.4 (3)
C5A—C6A—N2A—C7A	171.41 (18)	C5B—C6B—N2B—C7B	−177.51 (19)
O1A—C6A—N2A—C8A	168.05 (18)	O1B—C6B—N2B—C8B	−174.14 (19)
C5A—C6A—N2A—C8A	−11.9 (3)	C5B—C6B—N2B—C8B	5.0 (3)
C6A—N2A—C7A—O2A	−176.81 (19)	C6B—N2B—C7B—O2B	174.0 (2)
C8A—N2A—C7A—O2A	6.3 (3)	C8B—N2B—C7B—O2B	−8.4 (3)
C6A—N2A—C7A—N3A	3.2 (3)	C6B—N2B—C7B—N3B	−7.3 (3)
C8A—N2A—C7A—N3A	−173.61 (17)	C8B—N2B—C7B—N3B	170.29 (18)
O2A—C7A—N3A—C14A	−2.6 (3)	O2B—C7B—N3B—C14B	−5.6 (4)
N2A—C7A—N3A—C14A	177.31 (18)	N2B—C7B—N3B—C14B	175.8 (2)
C6A—N2A—C8A—C9A	111.0 (2)	C6B—N2B—C8B—C13B	80.3 (2)
C7A—N2A—C8A—C9A	−72.0 (2)	C7B—N2B—C8B—C13B	−97.5 (2)
C6A—N2A—C8A—C13A	−67.6 (2)	C6B—N2B—C8B—C9B	−94.0 (2)
C7A—N2A—C8A—C13A	109.5 (2)	C7B—N2B—C8B—C9B	88.2 (2)
C13A—C8A—C9A—C10A	0.6 (3)	C13B—C8B—C9B—C10B	−1.1 (3)
N2A—C8A—C9A—C10A	−177.96 (18)	N2B—C8B—C9B—C10B	173.11 (19)
C8A—C9A—C10A—C11A	0.2 (3)	C8B—C9B—C10B—C11B	−0.4 (3)
C9A—C10A—C11A—C12A	−0.5 (3)	C9B—C10B—C11B—C12B	1.2 (4)
C10A—C11A—C12A—C13A	0.0 (3)	C10B—C11B—C12B—C13B	−0.6 (4)
C9A—C8A—C13A—C12A	−1.1 (3)	C9B—C8B—C13B—C12B	1.7 (3)
N2A—C8A—C13A—C12A	177.48 (18)	N2B—C8B—C13B—C12B	−172.6 (2)
C11A—C12A—C13A—C8A	0.8 (3)	C11B—C12B—C13B—C8B	−0.9 (4)
C7A—N3A—C14A—C19A	−13.1 (3)	C7B—N3B—C14B—C15B	−174.3 (2)
C7A—N3A—C14A—C15A	168.4 (2)	C7B—N3B—C14B—C19B	6.1 (4)
C19A—C14A—C15A—C16A	−1.3 (3)	C19B—C14B—C15B—C16B	−2.7 (4)
N3A—C14A—C15A—C16A	177.3 (2)	N3B—C14B—C15B—C16B	177.6 (2)
C14A—C15A—C16A—C17A	0.5 (3)	C14B—C15B—C16B—C17B	0.7 (4)
C15A—C16A—C17A—C18A	0.6 (3)	C15B—C16B—C17B—C18B	1.5 (5)
C16A—C17A—C18A—C19A	−1.0 (3)	C16B—C17B—C18B—C19B	−1.8 (5)
C15A—C14A—C19A—C18A	0.9 (3)	C17B—C18B—C19B—C14B	−0.2 (5)
N3A—C14A—C19A—C18A	−177.53 (19)	C15B—C14B—C19B—C18B	2.5 (4)
C17A—C18A—C19A—C14A	0.2 (3)	N3B—C14B—C19B—C18B	−177.9 (3)

### Hydrogen-bond geometry (Å, º)

*Cg1*is the centroid of the C14*A*–C19*A* phenyl ring.

**Table:**

*D*—H···*A*	*D*—H	H···*A*	*D*···*A*	*D*—H···*A*
N3*A*—H3*E*···O1*A*	0.85 (3)	1.83 (3)	2.564 (2)	144 (3)
N3*B*—H3*F*···O1*B*	0.90 (3)	1.80 (3)	2.584 (2)	144 (2)
C19*A*—H19*A*···O2*A*	0.95	2.29	2.880 (2)	120
C19*B*—H19*B*···O2*B*	0.95	2.30	2.900 (3)	120
C9*A*—H9*A*···O1*A*^i^	0.95	2.46	3.378 (3)	161
C23*A*—H23*A*···*Cg*1^ii^	1.00	2.51	3.498 (8)	172
C23*B*—H23*B*···*Cg*1^ii^	1.00	2.47	3.43 (2)	161

Symmetry codes: (i) −*x*, −*y*, −*z*+1; (ii) *x*+1, *y*+1, *z*.
